# Adverse Events of Interferon Beta-1a: A Prospective Multi-Centre International ICH-GCP-Based CRO-Supported External Validation Study in Daily Practice

**DOI:** 10.1371/journal.pone.0026568

**Published:** 2011-10-25

**Authors:** Peter Joseph Jongen, Christian Sindic, Evert Sanders, Stanley Hawkins, Wim Linssen, Erik van Munster, Stephan Frequin, George Borm, and the Functional composite and quality of Life in Avonex-treated Relapsing multiple sclerosis patients (FLAIR) Study Group

**Affiliations:** 1 MS4 Research Institute, Nijmegen, The Netherlands; 2 Service de Neurologie, Cliniques Universitaires St. Luc U.C.L., Brussels, Belgium; 3 Department of Neurology, Amphia Hospital, Breda, The Netherlands; 4 Northern Ireland Regional Neurology Service, Royal Victoria Hospital, Belfast, Northern Ireland, United Kingdom; 5 Department of Neurology, St. Lucas Andreas Ziekenhuis, Amsterdam, The Netherlands; 6 Department of Neurology, Jeroen Bosch Ziekenhuis, 's-Hertogenbosch, The Netherlands; 7 Department of Neurology, St. Antonius Ziekenhuis, Nieuwegein, The Netherlands; 8 Department of Epidemiology, Biostatistics and HTA, Radboud University Nijmegen Medical Centre, Nijmegen, The Netherlands; Institute Biomedical Research August Pi Sunyer (IDIBAPS) - Hospital Clinic of Barcelona, Spain

## Abstract

**Background:**

Due to methodological shortcomings the available post-registration data on the adverse events (AEs) occurring in interferon beta-1a (INFb-1a)-treated patients fail to adequately validate phase III data and only partially inform on safety in daily practice. We assessed AEs in relapsing remitting multiple sclerosis (RRMS) patients treated with intramuscular (IM) INFb-1a in daily practice using data quality assurance measures similar to those in phase III trials.

**Methods:**

A prospective, International Conference on Harmonization (ICH) - Good Clinical Practice (GCP)-based, clinical research organization (CRO)-supported study in 36 practices in the Netherlands, Belgium, the United Kingdom and Luxembourg. During 24 months after start of IM INFb-1a treatment 275 RRMS patients were assessed for AEs' severity (mild, moderate, severe) and relationship to treatment (not, unlikely, likely, definite). Data were compared with those reported in the pivotal phase III trial.

**Findings:**

75.3% of the patients experienced one or more AEs that were likely or definitely related to INFb-1a. Of all AEs 40.5% were likely or definitely treatment-related; 68.5% of these were mild, and 3% severe. 6.6% of the patients discontinued treatment because of an AE. Compared to the pivotal phase III trial, we found statistically significantly lower incidences for most of the common AEs: headache, muscle ache, fatigue, fever, chills, nausea. One patient died following two cerebral vascular events in study month 22, both AEs were assessed as not related to INFb-1a.

**Conclusion:**

Three out of four RRMS patients treated with IM INFb-1a in daily practice experience treatment-related AEs, most of these being mild. Our data externally validate the favorable phase III safety profile of IM INFb-1a and suggest that the real-life incidence of treatment-related AEs is less than reported in the pivotal phase III trial. Larger studies are needed to detect rare, potentially hazardous AEs of IM INFb-1a.

## Introduction

Multiple sclerosis (MS) is a chronic, inflammatory, demyelinating disorder of the central nervous system (CNS) characterized by increasing disability [1]. Approximately 85% of the patients are initially diagnosed with relapsing-remitting MS (RRMS), which is characterized by partial or complete recovery after relapses [1], [2]. Interferon beta-1a (INFb-1a), administered intramuscular (IM) once weekly in a dose of 30 microgram (Avonex®), is a first-line disease modifying drug (DMD) for the treatment of patients with RRMS. A phase III randomized placebo-controlled trial demonstrated the efficacy of IM INFb-1a in reducing relapses and relapse-related disability, with mild to moderate adverse events (AEs) [3]. Symptoms reported more frequently by IM INFb-1a-treated than by placebo-treated patients were headache, flu-like symptoms, muscle aches, nausea, fever, asthenia, chills and diarrhoea [3].

Phase III trials of two years duration in limited numbers of patients cannot fully inform on AEs. After registration of a drug phase IV studies are needed for external validation and for the detection of rare and long-term AEs. Yet, most post-registration studies on IM INFb-1a only partially addressed safety aspects [4]–[9]. Moreover, studies were often single-centred, restricted to academic settings, performed in one region or country, or retrospective [4]–[12]. Some studies also included patients treated with subcutaneous INFb-1a or INFb-1b, with numbers too small to enable informative subgroup analysis [13], [14]. As a result, the available post-registration data on the safety of IM INFb-1a fail to adequately confirm phase III data and only partially inform on AEs in daily practice.

Phase III trials are strictly conducted according to the International Conference of Harmonization (ICH) guidelines on Good Clinical Practice (GCP) and involve clinical research organizations (CROs) [15]. The use of standardised and detailed clinical research files (CRFs) and standard operating procedures (SOPs), and regular monitoring contribute to consistency, completeness, and overall quality of AE data. In contrast, phase IV studies are not likely to engage CROs, use detailed CRFs or explicitly monitor the application of ICH-GCP guidelines. Due to these methodological differences the assessments of AEs in phase IV studies may lack the quality that is needed for adequate validation of phase III data, and are most likely incomplete. At the same time there is an obvious need of detailed and quantitative information on AEs in real life, as patients and doctors base their choices between treatment options on weighing benefits against risks [16].

To our knowledge, prospective, ICH-GCP-based, CRO-supported studies on AEs in patients treated with IM INFb-1a in daily practice have not been performed. The FLAIR (Functional composite and quality of Life in Avonex-treated Relapsing multiple sclerosis patients) study was designed to prospectively evaluate health-related quality of life (HRQoL), disability and AEs in RRMS patients treated with IM INFb-1a in real life settings. Two-hundred-eighty-four patients were included in 36 hospitals and specialised MS centres in the Netherlands, Belgium, the United Kingdom and Luxemburg. HRQoL and disability in these patients have been reported previously [17]. Here we present the AE results of the study.

## Results

### Patients

A total of 284 patients were included. In the Netherlands 151 patients (17 sites), Belgium 117 patients (16 sites), the U.K. 15 patients (two sites), and Luxemburg (one patient, one site). Eighty-nine patients were male (31%), 195 female (69%). Mean age was 38.6 years (SD 10.1), mean disease duration 6.6 years (Standard Deviation [SD] 6.6), mean pre-study annualized relapse rate 2.2 (SD 1.0), and mean baseline EDSS score 2.4 (SD 1.2). Two-hundred-and-four patients (71.8%) completed the study, whereas 80 (28.2%) discontinued prematurely. Reasons for discontinuation were: AE(s) in 22 (7.7%), non-compliance in 4 (1.4%), withdrawal of consent in 28 (9.9%), lost to follow-up in 9 (3.2%), relapse in 9 (3.2%), investigator's decision in 1 (0.4%), and other reason in 7 patients (2.5%). Mean time to discontinuation was 329.7 (SD 224.7) days. A total of 275 patients (96.8%) were included in the safety analysis set.

### Adverse events overall

A summary of AEs is shown in Table 1. Two-hundred-forty-five (89%) patients experienced one or more AEs, and 207 patients (75.3%) experienced one or more AEs that were classified as definitively or likely related to INFb-1a. More than 40% of all AEs were considered definitively or likely related to INFb-1a. AEs likely related to INFb-1a were reported by 125 patients (45.5%), and definitely related AEs by 120 (43.6%).

**Table 1:** Overview of adverse events in 275 patients from the safety analysis set.

		Patients		Events	
		N	%	n	%
Any AE*	All	245	89.1	967	100.0
	Not related†	193	70.2	579	59.9
	Related‡	207	75.3	388	40.1
	Non-serious	241	87.6	865	89.5
	Serious	53	19.3	97	10.0
	Deaths	1	0.4	2#	0.2
According to severity*	Missing	5	1.8	8	0.8
	Mild	208	75.6	625	64.6
	Moderate	126	45.8	293	30.3
	Severe	31	11.3	41	4.2
According to relationship*	Missing	3	1.1	5	0.5
	No	161	58.5	446	46.1
	Unlikely	71	25.8	128	13.2
	Likely	125	45.5	210	21.7
	Definite	120	43.6	178	18.4
AEs leading to withdrawal	All	22	8.0	38	3.9
	No or unlikely related	6	2.2	9	0.9
	Likely or definite related	18	6.5	29	3.0

N, number of patients; n, number of AEs; AE, adverse event;

*, patients could appear in more than one category;

† , not related, unlikely related or assessment missing;

‡ , likely related or definitely related;

# , two AEs in one patient were reported as leading to death.

The majority of all AEs were mild (64.6%), 30.3% was moderate and only 4.2% severe. Table 2 shows the numbers and percentages of mild, moderate, and severe AEs according to relationship to INFb-1a treatment. The majority of likely or definitely related AEs were mild, and related AEs that qualified as severe were rare.

**Table 2:** Overview of numbers and percentages of mild, moderate, and severe adverse events according to relationship to INFb-1a treatment.

	Mild	Moderate	Severe	Total
Not related	298	124	22	444
	(31.1%)	(12.9%)	(2.3%)	(46.4%)
Unlikely related	59	59	7	125
	(6.2%)	(6.2%)	(0.7%)	(13.1%)
Likely related	152	55	4	211
	(15.9%)	(5.7%)	(0.4%)	(22.0%)
Definitely related	114	56	8	178
	(11.9%)	(5.9%)	(0.8%)	(18.6%)
Total	623	294	41	958
	(65.0%)	(30.7%)	(4.3%)	(100%)

In 22 patients (8.0%) AEs led to withdrawal from the study, in 18 (6.6%) the AE was considered related to INFb-1a.

### Adverse events by body system

Table 3 presents the most often reported related AEs, occurring in at least 4% of the patients. The body system most often affected by related AEs was ‘body as a whole’. The most often reported related AEs were, by decreasing frequency, flu-like symptoms, headache, muscle pain, injection site pain, fever, fatigue, and depression.

**Table 3:** Most common likely or definitely INFb-1a-related adverse events by body system in 275 patients.

Body system* †	Patients		Events	
	N	%	n	%
Body as a whole	197	71.6	277	71.4
*Chills*	8	2.9	8	2.1
*Fatigue*	11	4.0	11	2.8
*Fever*	11	4.0	14	3.6
*Flu-like symptoms*	144	52.4	150	38.7
*Headache*	36	13.1	37	9.5
*Injection site pain*	18	6.5	19	4.9
*Malaise*	5	1.8	6	1.5
*Pain*	4	1.5	4	1.0
Digestive system	10	3.6	11	2.8
*Nausea*	5	1.8	5	1.3
Musculoskeletal system	23	8.4	28	7.2
*Muscle ache*	20	7.3	23	5.9
Nervous system	40	14.5	45	11.6
*Depression*	11	4.0	11	2.8
*MS aggravation*	4	1.5	4	1.0
*Sleep disturbance*	5	1.8	5	1.3
Skin and appendages	10	3.6	10	2.6

N, number of patients; n, number of events;

*, in at least 4 patients; totalling 371 (95.6%);

† , patients could have events in more than one category.

### Serious adverse events

A total of 97 SAEs were experienced by 53 patients (19.2%) and pertained mostly to the nervous system. SAEs occurring in more than one patient are presented in Table 4. The most often reported SAE was ‘MS aggravation’. No other SAE was reported in more than three patients (<1%). Five patients (1.8%) reported a total of seven SAEs considered as likely related to INFb-1a: depression, suicide attempt (two patients), MS aggravation (two patients), gait disturbance and dystonia. Three SAEs - both suicide attempts and one depression – resulted in discontinuation of INFb-1a. No SAEs were classified as definitely related to INFb-1a. One female patient, aged 58 years at baseline, died following a cerebral vascular attack on study day 650 and a cerebral venous thrombosis (sinus thrombosis) on study day 652. Both AEs were assessed as not related to INFb-1a.

**Table 4:** Serious adverse events occurring in more than one patient by body system in 275 patients.

Body system	Patients		Events
	N	%	n
Body as a whole	6	2.2	8
*Injury*	2	0.7	2
*Suicide attempt*	2	0.7	2
Cardiovascular system	2	0.7	2
Digestive system	4	1.5	5
*Colitis*	2	0.7	2
Nervous system	41	14.9	68
*Depression*	2	0.7	2
*Gait disturbance*	2	0.7	2
*MS aggravation*	35	12.7	47
*Paresthesia*	3	1.1	3
*Walking difficulty*	3	1.1	3
Special senses	4	1.5	4
*Optic neuritis*	2	0.7	2
Urogenital system	4	1.5	5

N, number of patients; n, number of events; %, percentage based on N = 275.

### Discontinuation due to adverse events

A total of 38 AEs in 22 patients led to treatment discontinuation. Twenty-nine AEs leading to discontinuation were assessed as likely or definitively related to INFb-1a. The most common of these were ‘flu-like symptoms’ (eight events), ‘depression’ and ‘headache’ (three events each). Three likely related AEs leading to withdrawal were SAEs (two suicide attempts and one depression). AEs leading to discontinuation in more than one patient are given in Table 5, the most often reported AEs being ‘flu-like symptoms’ and ‘MS aggravation’.

**Table 5:** Adverse events leading to withdrawal in more than one patient presented by body system in 275 patients.

Body system	Patients		Events
	N	%	n
Body as a whole	14	5.1	20
*Fatigue*	2	0.7	2
*Flu-like symptoms*	9	3.3	9
*Headache*	3	1.1	3
*Suicide attempt*	2	0.7	2
Musculoskeletal system	2	0.7	2
*Muscle pain*	2	0.7	2
Nervous system	10	3.6	13
*Depression*	3	1.1	3
*MS aggravation*	4	1.5	4

N, number of patients; n, number of events.

## Discussion

Clinicians and patients need detailed and quantified information on incidence and severity of AEs as observed in real life [18]. In phase III trials selected patients are treated in settings not representative of daily practice, and for these data to be clinically useful they must be externally validated [19]. In spite of IM INFb-1a being registered for RRMS in Europe in 1998, post-registration data that adequately validate the safety profile reported in the pivotal phase III trial are fragmentary.

With application of data quality assurance measures similar to those in phase III trials, we assessed AEs in real life settings representative of daily MS care in different countries. Over a 2 year period 75.3% of the patients had one or more (mean 1.8) AEs that were likely or definitely related to INFb-1a, which makes that 40.1% of all health changes were considered treatment-related. Most of the related AEs were mild, severe ones were infrequent, and only rarely (3.0%) did related AEs result in treatment discontinuation. We previously observed in this patient group that IM INFb-1a treatment was associated with an increase in mean HRQoL. Although three-quarters of the patients reported mild AEs, moderate ones were seen in less than half, and severe AEs only in one in nine patients. It may be hypothesized that overall in the study group AEs had no major impact on wellbeing and that any impact was outweighed by perceived benefits e.g. a decrease in relapses.

The demographic and neurological characteristics of the patients in the present study were similar to those of the INFb-1a-treated group in the pivotal phase III trial (mean age 36.7 [SD 7.2] years, mean disease duration 6.6 [SD 5.8] years, mean baseline EDSS score 2.4 [SD 0.8]) [3]. This may relate to the fact that in the countries where we performed our study the registered indication of IM INFb-1a and the criteria for reimbursement are actually based on the phase III inclusion criteria. Overall, the most frequently occurring AEs in our patients were similar to those reported by Jacobs et al. [3]. Interestingly, however, for the majority of these AEs the incidences we observed were significantly lower: headache (19.3% vs. 67%), muscle ache (15.6% vs. 34%), fatigue (12.7% vs. 21%), fever (5.1% vs. 23%), nausea (4.7% vs. 31%), and chills (2.9% vs. 21%); the differences in injection site reactions and depression could not be calculated, as the paper by Jacobs et al. only reported the ranges (Table 6). Thus, apart from flu-like symptoms, the various major AEs were 3.6 to 17.2 times less frequent in our patient population than in the INFb-1a-treated phase III group. This discrepancy may relate to the fact that the setting of a registration study facilitates AE reporting, as doctors and nurses have ample time to communicate with patients, whereas in daily practice there may be a bias towards reporting of those AEs that are thought of as clinically relevant. Moreover, patients participating in phase III studies might be more motivated to report any change in health status. Differences in patients' motivation and communication with caregivers may also explain why 6.6% of our patients discontinued treatment due to INFb-1a-related AEs, in contrast with only one INF-b-1a-treated patient (<1%) in the phase III study who discontinued treatment because of an AE [3].

**Table 6:** Adverse events reported more frequently by INFb-1a-treated than placebo-treated patients in the pivotal IM INFb-1a phase III study (Jacobs et al. 1996) compared to frequency of similar adverse events in the present study.

	Jacobs et al. Phase III		FLAIR study Phase IV			
		All AEs		Related AEs	
	N	%	N	%	N	%
Chills	33	21	8	2.9**	8	2.9**
Fatigue†	33	21	35	12.7*	16	5.8**
Fever	37	23	14	5.1**	11	4.0**
Flu-like symptoms	96	61	145	52.7	144	52.4
Headache	106	67	53	19.3**	36	13.1**
Nausea	49	31	13	4.7**	5	1.8**
Muscle ache‡	53	34	43	15.6**	20	7.2**
Pain			14	5.1	4	1.5
Injections site reactions#		10–15	18	6.5	18	6.5
Depression		10–15	28	10.2	11	4.0

N, number of patients;

† , asthenia in Jacobs et al.; fatigue and malaise in INFb-1a-related AEs in FLAIR patients;

‡ , including muscle pain and myalgia in FLAIR patients;

# , injection site pain in FLAIR patients;

*, P<0.05;

**, P<0.0001. Differences in frequencies of injection site reactions and depression were not statistically tested.

It is unlikely that the symptomatic treatment of AEs in our study explains the lower incidences. We advised patients acetaminophen 625–1000 mg/day Q 4 hours and if necessary a NSAID for treatment of influenza-like symptoms, whereas neurologists were advised to treat fatigue with amantadine and spasticity with baclophen, when deemed necessary.

Likewise, during the pivotal phase III study acetaminophen, 650 mg, was given prior to and for 24 hours after each injection, and patients received appropriate medical care, including antidepressants and antispastic drugs [3].

To our knowledge two studies specifically investigated IM INFb-1a-related AEs in daily practice [10], [11]. Both were performed in academic hospitals and included 27 and 96 patients, respectively. Reports did not inform on measures assuring data quality. As the terms to denominate AEs and ways of presenting data vary between studies comparisons are difficult. Fernandez et al. reported on safety in RRMS patients, treated with IM INFb-1a in three centers over a period of 2 years (Table 7). Incidences of asthenia (45.1%) and fever (42.9%) were remarkably higher than found in the dose-comparing phase IIIb by Clanet et al. and in our study [20]. The incidence of depression was in the same range as in our patients (10%), but in only 4% we considered depression treatment-related; and in the phase IIIb study depression was reported by 35% of the patients in the 30 µg group [20] (Table 7). In fact, it may be difficult for patients and doctors to differentiate symptoms relating to INFb-1a from similar MS symptoms (asthenia, fatigue, depression, muscle aches) or from health changes that are frequent in the general population (headache). In long duration trials, most patients will report at least one AE and making sense of common, reversible and minor AEs is difficult. Assessment of any relationship between AEs and INFb-1a also depends on the experience doctors and patients have with both MS and INFb-1a. Cultural factors may also be at play. Yet, for four of the five most common AEs the incidence found in our study corresponded with the estimates given in the SPC and package leaflet texts.

**Table 7:** Incidences of most common adverse events as reported in the pivotal phase III study (Jacobs et a. 1996), the dose-comparing phase IIIb study (Clanet et al. 2002), an observational phase IV study (Fernandez et al. 2003), the SPC text, and as found in the present study.

	Jacobs et al.	Clanet et al.	Fernandez et al.	SPC text	FLAIR study	
					All AEs	Related AEs
Year	1996	2002	2003			
Phase	III	IIIb	IV		IV	IV
N	193	402	96		284	284
Headache	67%	28%	<10%	>10%	19%	13%
Flu-like syndrome	61%	85%	45%	>10%	53%	52%
Fever	23%		43%	>10%	5%	4%
Asthenia/fatigue	21%		>45%	<10%	13%	6%
Depression	n.m.f.	35%	10%	<10%	10%	4%

SPC, Summary of Product Characteristics; n.m.f., not more frequent than in placebo-treated patients (Jacobs et al. 1996).

SAEs, none of which were attributed to treatment, occurred in 12 patients (6.2%) in the INFb-1a-treated phase III study group [3], whereas in the present study 1.8% of the patients reported seven treatment-related SAEs: suicide attempt, depression and worsening of neurological symptoms; of which two suicide attempts and one depression resulted in discontinuation of INFb-1a. A transient increase in spasticity following dosing with INFb has been reported in MS patients [21], [22]. A temporary worsening of MS symptoms may also relate to INFb-1a-induced fever. The absence of treatment-related SAEs in the pivotal phase III trial may reflect a more restrictive patient screening procedure.

One of the patients died from a cerebral vascular attack and a sinus thrombosis 21 months after start of INFb-1a, both AEs were considered not treatment-related. Jacobs et al. reported that one INFb-1a recipient died from pulmonary embolism and cardiac arrhythmia designated as unrelated to the study drug [3]. These observations may justify the question whether vascular events are a rare side effect of INFb. On the other hand, factors like steroid use, decreased mobility or associated medical conditions may also increase the risk of vascular events in RRMS patients. Stroke after initiation of INFb has been reported in a patient with RR white matter disease, who turned out to have a primary angiitis of the CNS [23]. Only prospectively acquired data in large patient groups can answer this question.

This study has several limitations. Its size and duration preclude detection of rare AEs or AEs that develop in the long term. As we performed no laboratory assessments, subclinical changes have gone undetected, although from a clinical point of view these changes may be thought of as of minor importance. We also did not differentiate between AEs occurring in the 1^st^ and the 2^nd^ year of treatment. Incidences of AEs may change during a study [24], and it has been known that INFb-related AEs typically start in the first weeks and mostly subside within 3 to 6 months. In a 12-month study in 27 RRMS patients who had started treatment with IM IFNb-1a, the percentage of patients with AEs remained constant, but the mean number of AEs increased significantly from the 6th to the 12th month with significant changes over time for almost all AEs [10].

In conclusion, when using phase III performance standards we observed that 75.3% of RRMS patients who were treated for up to 2 years with IM INFb-1a in daily practice experienced one or more (mean 1.8) treatment-related AEs. Most of these AEs were mild, severe ones were rare, and 6.6% of the patients discontinued INFb-1a because of a related AE. For the majority of the most common treatment-related AEs the incidences were in accordance with the SPC text. However, when compared to the pivotal phase III trial we observed significantly lower incidences for most of the treatment-related AEs [3].

## Methods

### Ethics statement

The decision to start IM INFb-1a was preceded by and independent from the decision to inform patients about the study. Written informed consent was obtained prior to any study-related procedure. The study was conducted in accordance with the Declaration of Helsinki (South Africa 1996), the ICH-GCP guidelines, and in compliance with national drug laws [15], [25]. The protocol was submitted to the Independent Review Board (IRB), an approved ethical committee residing in Amsterdam, the Netherlands. The IRB concluded that, because of the observational design of the study, a review by an ethical committee was not required, as the study did not qualify for being tested according to the Dutch Medical Research Involving Human Subjects Act of 1999 [25]. Subsequently the ethical committees at all institutions and hospitals where participants were recruited were informed by the local investigators about the IRB's judgment and the study protocol was not reviewed by the local ethical committees.

### Study design and administrative procedures

An investigator-initiated (PJJ), prospective, multi-centre, international, observational study, involving 46 neurologists in 36 practices in the Netherlands, Belgium, the United Kingdom and Luxembourg. Twenty-two practices were in general hospitals, 10 in university hospitals and 4 in independent MS clinics. Study duration was 24 months.

ClinicalTrials.gov identifier NCT00534261. The CRO Kendle International was responsible for project management, monitoring, data management and statistical analysis. A steering committee provided scientific and medical direction and oversaw the administrative progress. A patient was considered to be enrolled once a baseline evaluation had been completed. Subjects that withdrew before completion were followed-up, within 2 weeks after discontinuation, as per month 24 and were not replaced. The first patient entered the study on 4 November 1999 and the last patient completed the study 11 February 2004.

IM INFb-1a was prescribed by the treating neurologist. Commercially available drug was dispensed by the pharmacist as per regular care and reconstituted and administered once a week IM according to product instructions. Patients were instructed how to inject and were offered a service provided by trained registered nurses. The nurses visited patients at home and trained them or their care-partners on how to administer.

Three protocol amendments were implemented after the protocol was finalized on May 1999. The information in this paper reflects the situation after Amendment 3. Changes regarding safety were: No laboratory parameters were to be determined or analyzed for this study (Amendment 1, dated 28 July 1999), and serious adverse events (SAEs) monitoring and reporting were specified (Amendment 2, dated 10 March 2000).

### Eligibility criteria

Inclusion criteria were: 1) RRMS, 2) age between 18 and 70 years, inclusive, 3) two relapses in the last 24 months, 4) disease duration of at least 12 months, 5) expanded disability status scale (EDSS) score of 5.5 or less, 6) naïve with respect to INFb treatment. Criteria 1 to 5 were according to the Summary of Product Characteristics (SPC) and reimbursement policies. Exclusion criteria were: 1) history of any significant cardiac, hepatic, pulmonary or renal disease; immune deficiency; or other medical conditions that would preclude therapy with INFb, 2) history of severe allergic or anaphylactic reactions or history of hypersensitivity to human albumin, 3) history of seizures within the previous 3 months, 4) history of intolerance to INFs, 5) female patients who were pregnant or breast feeding, 6) for female subjects, unwillingness to practice effective contraception, as defined by the investigator, unless postmenopausal or surgically sterile; women considering becoming pregnant during the study period of 24 months were to be excluded, 7) previous participation in this study, 8) history of intolerance to acetaminophen (paracetamol), naproxen or other non-steroidal anti-inflammatory drugs (NSAIDs), that would preclude use of at least one of these drugs. Criteria 1 to 6 were according to the SPC text.

### Adverse events assessment and management

AEs were defined as any sign or medical diagnosis noted by medical personnel or symptom reported by the patient, regardless of relationship to treatment, that: 1) started any time after start of treatment, whereby signs, symptoms and diagnoses occurring prior to the first dose of INFb-1a were not considered to be AEs if they did not increase in severity; or 2) had worsened when the event had been present prior to the first dose of INFb-1a. AEs were assessed at months 3, 6, 12, 18, and 24.

All AEs reported by the patient or observed by study site personnel were recorded in the patient's CRF. For patients who prematurely withdrew from the study AEs were collected within 2 weeks after withdrawal. In addition, for patients who discontinued because of reasons related to INFb-1a AEs were followed up until they had resolved.

The AE information recorded included severity - mild, moderate, severe- and relationship to INFb-1a - unrelated, unlikely related, likely related, definitely related. Mild: Symptom(s) barely noticeable to subject or does not make subject uncomfortable; does not influence performance or functioning; prescription drug not ordinarily needed for relief of symptom(s) but may be given because of personality of subject. Moderate: Symptom(s) of a sufficient severity to make subject uncomfortable; performance of daily activity is influenced; subject is able to continue in study; treatment for symptom(s) may be needed. Severe: Symptom(s) cause severe discomfort; severity may cause cessation of treatment with study drug; treatment for symptom(s) may be given and/or subject hospitalized. For reason of analysis AEs that were assessed as ‘definitely related’ or ‘likely related’ were termed ‘related AEs’, and AEs assessed as ‘not related’ or ‘unlikely related’ and those with missing assessment were termed ‘not related’.

A serious AE (SAE) was defined as: 1) death, 2) life-threatening event, i.e. an event that places the subject, in the view of the investigator, at immediate risk of death from the event as it occurred; 3) event that requires or prolongs hospitalisation; 4) event that results in persistent or significant disability or incapacity; 5) congenital anomaly or birth defect diagnosed in a child of a patient; 6) event that necessitates medical or surgical intervention to prevent one of the outcomes listed above. SAEs that were unresolved at month 24 or at the time the patient discontinued INFb-1a treatment were followed up until the event had resolved or the clinical course had stabilised.

Patients were instructed on management of AEs related to INFb-1a according to Munschauer and Kinkel, 1997 [26]. Acetaminophen (paracetamol) 625–1000 mg/day Q 4 hours was advised for influenza-like symptoms, if necessary with changed time of administration or switch to a NSAID (ibuprofen, naproxen) with or without acetaminophen (paracetamol). To the discretion of the neurologist fatigue was to be treated with amantadine 100 mg twice daily, spasticity with baclophen 10–20 mg simultaneously with IM INFb-1a injection, then Q 4–6 hours as needed. AE management was recorded in the CRF.

### Data quality assurance and statistics

Data quality was assured by monitoring and data management procedures. Each site was periodically monitored by a CRO representative to compare data collected in the CRF with the investigator's source document. Source document verification was 20%. Data management and entry were performed by the CRO. To ensure that entered data accurately reflected those contained in the CRF, double data entry was performed using TrialBase™. The value entered on first entry was compared to the value entered on second entry, with any differences flagged for later resolution. To achieve completeness and consistency of data automated edits and manual reviews were performed according to a validation plan prepared in cooperation with the Steering Committee. Queries were printed, forwarded to the site and resolved by the investigator. After transfer of data to a statistical package (SAS, version 8.2 [SAS Institute Inc. Cary NC, USA, 1997]) data correctness was validated according to SOPs. Coded data and the codes were checked for correctness. No audits were performed.

The safety analysis set was defined as all subjects who received at least one dose of INFb-1a and had at least one post-baseline safety assessment.

AEs were summarized overall, and by severity and relationship to INFb-1a. The summary tables include incidence estimates for each overall system organ class and for individual events within each system organ class. Preferred terms and the COSTART dictionary version 5 were used to denominate AEs.
